# Can P1NP Levels Influence Management Planning for Patients With a Fragility Hip Fracture Receiving Anti-resorptive Medications?

**DOI:** 10.7759/cureus.104983

**Published:** 2026-03-10

**Authors:** Mustafa Kraidi, Iain Wilkinson, Somaditya Bandyopadhyay

**Affiliations:** 1 Internal Medicine, Surrey and Sussex Healthcare NHS Trust, Redhill, GBR; 2 Geriatric Medicine, Surrey and Sussex Healthcare NHS Trust, Redhill, GBR; 3 Orthogeriatric Medicine, Surrey and Sussex Healthcare NHS Trust, Redhill, GBR

**Keywords:** anti-resorptive medications, biomarkers, bone turnover, fragility hip fracture, p1np

## Abstract

Background: The procollagen type 1 N-terminal propeptide (P1NP), a byproduct of type I collagen synthesis, is useful in clinically monitoring anti-resorption medications. The role of P1NP in anti-resorption therapy in older bisphosphonate-taking individuals who have suffered another fracture is unclear.

Objectives: This study aims to describe serum P1NP levels in patients aged ≥60 years who sustained a fragility neck of femur fracture while receiving osteoporosis therapy and to describe how P1NP results were associated with subsequent bone health management decisions, defined as documented decisions to continue, stop, or change osteoporosis therapy (including switch/escalation) and/or request additional investigations.

Methods: This retrospective descriptive cohort study, conducted between March 2017 and September 2021, involved patients aged 60 years or older who experienced intra- or extracapsular femoral neck fractures while receiving osteoporosis therapy and had serum P1NP assessed before surgery. Routinely collected data were extracted from departmental databases and systems accessible through the NHS computers at East Surrey Hospital as part of an ongoing quality improvement project.

Results: Out of the 2,303 total fractures during the study period, 58 patients (2.5%) had serum P1NP levels tested. The mean age was 84.6 ± 8.08 years, with a female-to-male ratio of 8.7:1; 34 (58.6%) had intracapsular and 24 (41.6%) had extracapsular types of fractures. Eighteen patients (31%) had P1NP levels of 40 ug/l or higher; six (10.3%) had P1NP levels between 36 and 39 ug/l, and 34 patients (58.6%) exhibited suppressed P1NP levels (below 35 ug/l). For those who had suppressed P1NP, five (55.6%) of the nine patients (who had been receiving treatment for over five years) had their treatment discontinued, two (22.2%) had their treatment plans modified because of DXA scan results, and two remained on the same treatment plan. Three patients on therapy for up to five years had P1NP levels above 40 ug/l owing to memory loss or inexperience with oral alendronate; therefore, adherence was low. Change to IV zoledronate or patient education was offered.

Conclusion: In this selected cohort, measuring pre-operative P1NP levels supported patient-centred multidisciplinary (MDT) bone health planning. Clinicians considered P1NP alongside DXA findings and the broader clinical context when documenting MDT post-fracture bone health plans, with management changes commonly recorded among patients receiving long-term therapy (>5 years). Notably, in this long-term treated group, suppressed P1NP commonly coincided with documented decisions to stop or adjust therapy. Given the retrospective design, small sample size, lack of a comparator group, and absence of outcome data, these findings provide real-world insight into current practice and may support development of a more standardised approach to incorporating P1NP into post-fracture bone health pathways.

## Introduction

Low-impact hip fractures (fragility fractures) in the elderly, often caused by falls, are a leading cause of admission to orthopedic and orthogeriatric units [1]. Data from the National Hip Fracture Database (NHFD) indicate that over 500,000 hip fracture cases were recorded between 2007 and 2017, while approximately 70,000-80,000 hip fractures occur annually in the United Kingdom [2]. These fractures are associated with substantial mortality rates; approximately 5% of patients with a hip fracture die within a month, and 30% die within a year [1]. Approximately £2 billion is spent annually in the United Kingdom on medical and social care for hip fractures [1]. To prevent these fragility fractures, primary and secondary strategies were established, including falls prevention strategies and promotion of osteoporosis treatment. These strategies are crucial for the current and future epidemiology of hip fractures.

The clinical guidelines from the National Institute for Health and Care Excellence (NICE) and the National Osteoporosis Guideline Group (NOGG) recommend monitoring treatment adherence and tolerance for patients starting oral anti-resorptive medications [1,3]. This should be done at intervals of approximately 12-16 weeks after starting treatment and then again after one year. After completing the treatment course, a final fracture risk assessment using the Fracture Risk Assessment Tool (FRAX) and a dual-energy X-ray absorptiometry (DXA) scan should be conducted. These recommendations do not incorporate biochemical markers of bone turnover (BTM) in the evaluation of fracture risk and monitoring therapy. Sheffield Teaching Hospitals has utilised BTM for more than 18 years to monitor osteoporosis medication in metabolic bone clinics [4].

The procollagen type 1 N-terminal propeptide (P1NP) is a serum-measurable BTM that shows promise as a useful tool for clinical practice evaluations [5]. P1NP is produced as a consequence of type 1 collagen biosynthesis during bone formation [4]. In addition to its value as a bone formation marker, P1NP may also be used to track the efficacy of anti-resorption drugs such as alendronate [6,7]. A substantial reduction in P1NP levels within a few months of initiating treatment suggests therapeutic efficacy [4]. In Sheffield Teaching Hospitals, P1NP has been the primary biomarker for more than seven years, and it is accessible from both primary and secondary care [8]. The current local guidelines mandate that patients undergo a baseline P1NP level assessment prior to commencing on anti-resorptive therapy, with a subsequent assessment occurring after six months [8]. The treatment's objective is to reduce bone turnover to a level that is associated with a low fracture risk, with a 10 µg/l reduction from baseline or a post-treatment level below 35 µg/l [8-10].

Since 2017, the orthogeriatric department at East Surrey Hospital has incorporated P1NP assessment for patients who have developed fragility hip fractures while receiving anti-resorptive treatment [11]. Although some studies have investigated the role of measuring P1NP to monitor osteoporosis treatment [5,8,9,12], the function of P1NP in formulating treatment plans for patients who present with fragility hip fractures while undergoing osteoporosis treatment has not yet been thoroughly investigated. Therefore, this study aimed to characterise P1NP levels in patients aged ≥60 years who sustained a fragility neck of femur fracture while receiving osteoporosis therapy, and to describe whether and how P1NP results were associated with subsequent bone health management decisions, defined as documented decisions to continue, discontinue, or change osteoporosis therapy (switch or escalation) and/or to undertake further investigation (e.g., DXA) in follow-up clinic correspondence.

## Materials and methods

This was a retrospective cohort analysis of clinical data obtained from orthogeriatric patients at East Surrey Hospital. Data were collected via departmental and hospital databases accessible through the NHS computer systems, including the Cerner software system for reviewing historical clinic letters and laboratory and imaging reports, the Picture Archiving and Communication System (PACS) for reviewing prior imaging, and the Advanced Patient-Centered Excellence (APEX) system for accessing historical laboratory results. In addition, archived clinic letters held within the department were reviewed.

Between March 2017 and September 2021, a cohort of 58 patients aged over 60 years who were admitted with intra- or extracapsular femoral neck fractures was identified. All included patients were receiving ongoing osteoporosis treatment, and serum P1NP levels were measured during the index hospitalisation. Patients were included if they met the following criteria: age ≥60 years, admission with a fragility femoral neck fracture while on ongoing osteoporosis treatment, and availability of a pre-operative P1NP result during the index admission.

P1NP was measured during the index admission and prior to surgical fixation in all included patients. P1NP testing was not routinely performed for all hip fracture admissions and was requested at the clinician's discretion as part of orthogeriatric bone health assessment in patients who sustained a fragility hip fracture despite established osteoporosis therapy, where assessment of bone turnover/treatment suppression and planning of post-fracture bone protection strategy were being considered.

Data collected included patient demographics (age and sex), anatomical fracture location (intracapsular or extracapsular), and the surgical fixation method employed. Available DXA scan records obtained before and after the fracture event were reviewed. Historical clinic correspondence was analysed to determine whether osteoporosis treatment plans had been modified, to identify relevant medical comorbidities, and to assess for the occurrence of any subsequent fragility fractures. Where available, plain radiographs were reviewed. P1NP values and their corresponding dates of assessment were also recorded.

Management decisions were made within routine multidisciplinary (MDT) practice. P1NP results were considered alongside DXA findings, duration of therapy, comorbidities, renal function, and adherence considerations. For this retrospective analysis, management decisions were extracted from clinical documentation. Where correspondence explicitly referenced the P1NP result as contributing to the decision, this was noted; otherwise, decisions were recorded as occurring after P1NP testing. Based on previous literature [13,14] and established reference ranges for human serum, a P1NP level of ≤35 µg/L was considered indicative of optimal suppression of bone turnover, while a level of ≥40 µg/L was considered elevated.

Statistical analyses were performed using the IBM SPSS Statistics for Windows, Version 25 (Released 2017; IBM Corp., Armonk, New York, United States). Continuous variables were expressed as mean, standard deviation (SD), or range, as appropriate. Group comparisons were conducted using the Mann-Whitney U test and Kruskal-Wallis test with Bonferroni post hoc correction. Categorical variables were expressed as frequencies and percentages and compared using the chi-square test or Fisher’s exact test, as appropriate. Statistical significance was defined as a two-sided p-value of <0.05.

## Results

A total of 58 patients were identified between March 2017 and September 2021 who had P1NP tested, accounting for 2.5% of the 2,303 total fractures during this period. The mean age of the patients was 84.6 ± 8.08 years, with a female-to-male ratio of 8.7:1, comprising 34 intracapsular and 24 extracapsular types of fractures. Details about the types of operation, osteoporosis treatment, and duration are illustrated in Table 1.

**Table 1:** Patient characteristics IC: intracapsular; EC: extracapsular; DHS: dynamic hip screw; IMN: intramedullary nail; HEMI: hemiarthroplasty; THR: total hip replacement; AA: alendronate; ZOL: zoledronate; DMAB: denosumab; RISI: risedronate; TERI: teriparatide

Characteristic		No	%
Sex	Female	52	89.7
	Male	6	10.3
Site	Left	31	53.4
	Right	27	46.6
Fracture type	IC	34	58.6
	EC	24	41.4
Operation	DHS	15	25.9
	IMN	9	15.5
	HEMI	29	50.0
	THR	5	8.6
Treatment	AA	51	87.9
	AA+ZOL	1	1.7
	DMAB	1	1.7
	Ibandronate	1	1.7
	ZOL	2	3.4
	RISI	2	3.4
Duration (year)	<1	17	29.3
	1-3	18	31.0
	3-5	11	19.0
	>5	12	20.7

**Figure 1:**
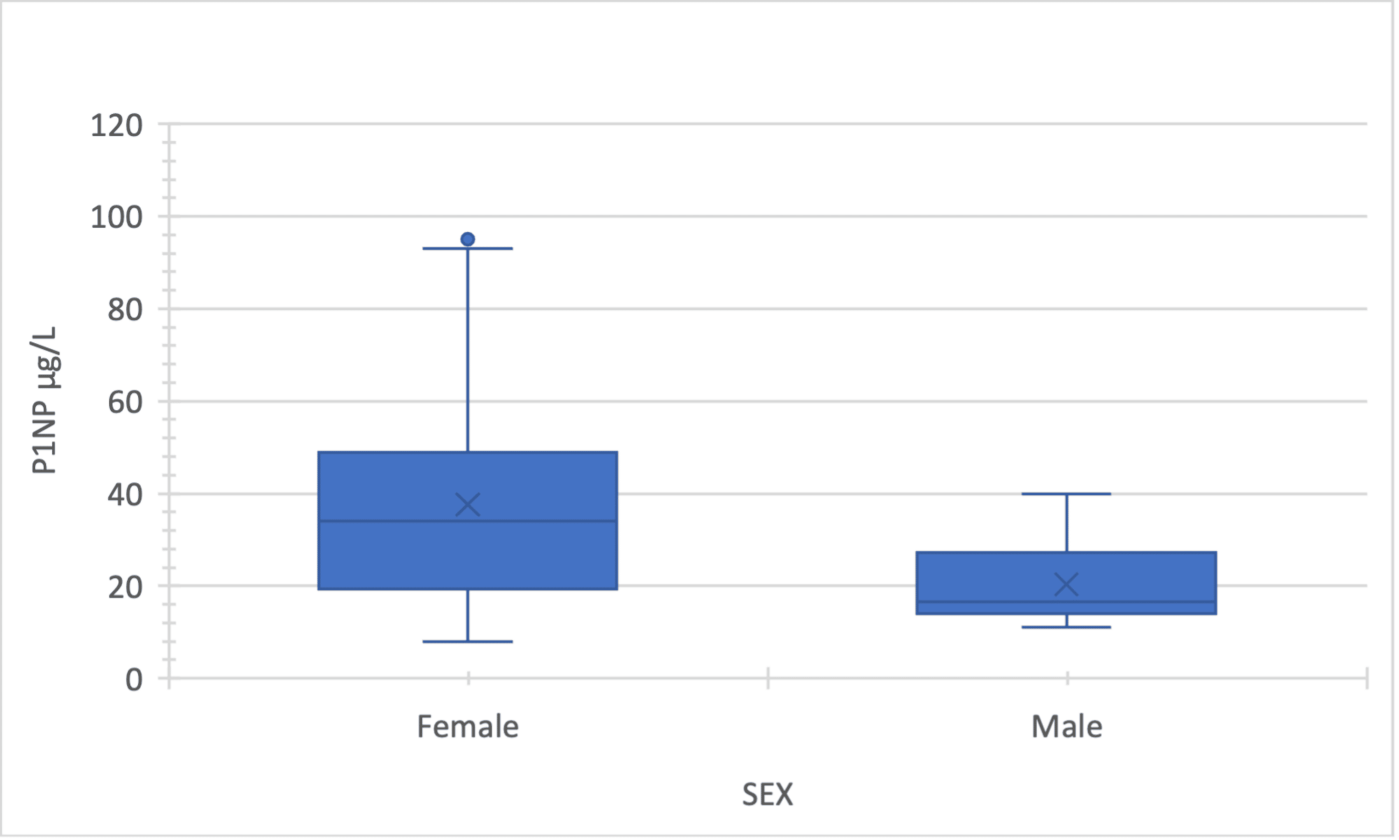
The level of P1NP according to the patient's gender P1NP: procollagen type 1 N-terminal propeptide Mann-Whitney test = 81.5; p = 0.056

The mean level of P1NP was 31 ± 22.9 µg/L, ranging between 8 and 96 µg/L. The level was lower in male compared to female patients (Mann-Whitney test = 81.5; p = 0.056) (Figure 1).

**Figure 2:**
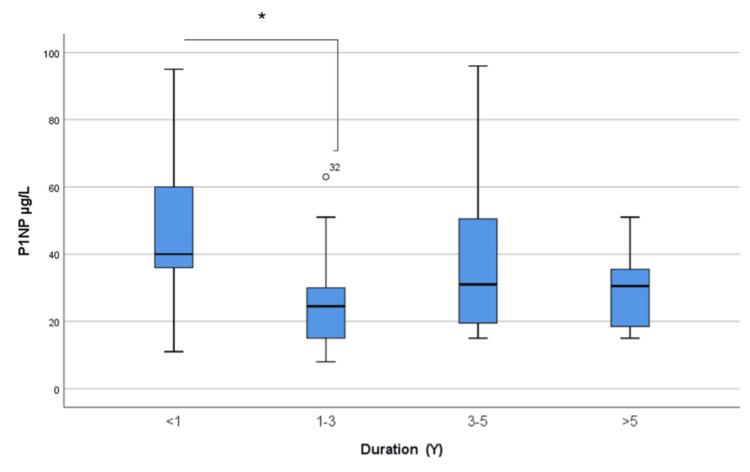
The level of P1NP according to the duration of osteoporosis treatment P1NP: procollagen type 1 N-terminal propeptide Kruskal-Wallis test = 8.614; p = 0.035. Pairwise comparisons adjusted by Bonferroni correction for multiple tests (p = 0.025)

Overall, 18 (31%) patients had P1NP levels of 40 µg/L or above; six patients (10.3%) had P1NP levels between 36 and 39 µg/L, and 34 (58.6%) patients had suppressed P1NP (≤35 µg/L). A significant difference was seen in the P1NP level of patients with different durations of treatment (Kruskal-Wallis test = 8.614; p = 0.035) (Figure 2). P1NP was significantly higher in those who were on the treatment for less than a year compared to those who were continuing on treatment for up to three years, with an adjusted Bonferroni post hoc p-value of 0.025. 

**Figure 3:**
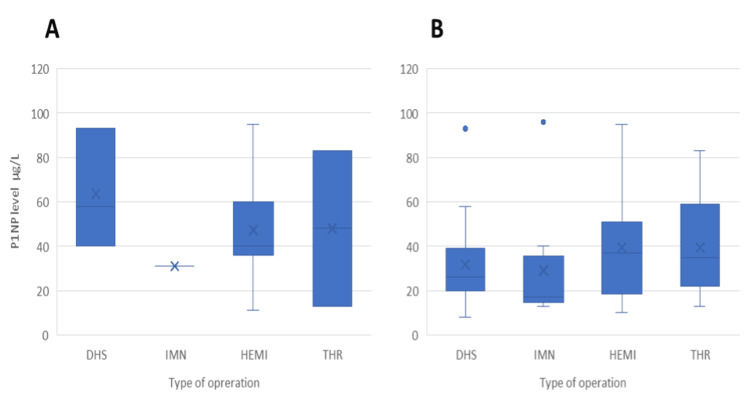
The level of P1NP according to operation type for (A) patients with less than a year of treatment and encountered the fracture (Kruskal-Wallis test = 2.145; p = 0.543), (B) all patients (Kruskal-Wallis test = 3.669; p = 0.300) P1NP: procollagen type 1 N-terminal propeptide; IC: intracapsular; EC: extracapsular; DHS: dynamic hip screw; IMN: intramedullary nail; HEMI: hemiarthroplasty; THR: total hip replacement

In patients with short-term treatment for less than a year (n = 17), the type of operation was further evaluated. As shown in Figure 3, patients who underwent the dynamic hip screw (DHS) procedure had a higher level of P1NP compared to other procedures; however, statistically, that was not significant (Kruskal-Wallis test = 2.145; p = 0.543). After all patients were assessed, the DHS procedure was not associated with higher P1NP levels (Kruskal-Wallis test = 3.669; p = 0.300).

**Figure 4:**
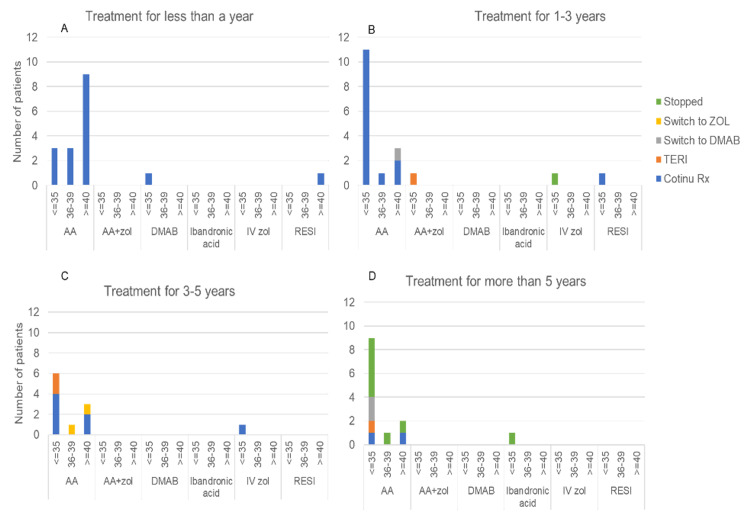
Modification of treatment plant according to P1NP levels (≤35, 36-39, and >40 µg/L) P1NP: procollagen type 1 N-terminal propeptide; AA: alendronic acid; ZOL: zoledronic acid; DMAB: denosumab; RESI: risedronate; TERI: teriparatide

The management decisions considering the P1NP level are illustrated in Figure 4 and Table 2. For those who had suppressed P1NP, five (55.6%) of the nine patients, those who had been receiving treatment for over five years, had their treatment discontinued. Two (22.2%) had their treatment plans modified because of DXA scan results, and two remained on the same treatment plan. Five of the seven patients who had been receiving treatment for three to five years remained on treatment, while two transitioned to teriparatide. One of the patients underwent an additional DXA scan. 

**Table 2:** Management plan in relation to P1NP level and duration of osteoporosis treatment P1NP: procollagen type 1 N-terminal propeptide; Rx: treatment; TERI: teriparatide; DMAB: denosumab; ZOL: Zoledro

Management plan	Duration of osteoporosis								Fisher's exact test	p-value
	<1 year		1-3 years		3-5 years		>5 years			
	No	%	No	%	No	%	No	%		
P1NP level ≤ 35 µg/L (n = 34)										
Continued Rx	4	100	12	85.7	5	71.4	1	11.1	19.141	0.001
Changed to TERI	0	0	1	7.1	2	28.6	1	11.1		
Switch to DMAB	0	0	0	0	0	0	2	22.2		
Switch to ZOL	0	0	0	0	0	0	0	0		
Stopped Rx	0	0	1	7.1	0	0	5	55.6		
P1NP level 36-39 µg/L (n = 6)										
Continued Rx	3	100	1	100	0	0	0	0	9.128	0.200
Changed to TERI	0	0	0	0	0	0	0	0		
Switch to DMAB	0	0	0	0	0	0	0	0		
Switch to ZOL	0	0	0	0	1	100	0	0		
Stopped Rx	0	0	0	0	0	0	1	100		
P1NP level ≥40 µg/L (n = 18)										
Continued Rx	10	100	2	66.7	2	66.7	1	50	33.739	<0.001
Changed to TERI	0	0	0	0	0	0	0	0		
Switch to DMAB	0	0	1	33.3	0	0	0	0		
Switch to ZOL	0	0	0	0	1	33.3	0	0		
Stopped Rx	0	0	0	0	0	0	1	50		

Of the 14 patients who were on treatment for one to three years, 12 (85.7%) remained on the same treatment plan, one had treatment discontinued, and the other had therapy switched to teriparatide. The identical treatment regimen was maintained by all four patients who had been receiving treatment for less than one year.

We endeavoured to determine the reason for P1NP levels of 40 µg/L or higher. Ten (55.6%) of the 18 patients who met this criterion had been on bisphosphonate treatment for less than one year. Despite the fact that one patient had P1NP levels of 80 ug/l and three patients had levels spanning from 90 to 93 ug/l, no modifications were made to the treatment plan for this subgroup. Additionally, we identified three (16.7%) patients with P1NP levels of 96, 70, and 62 µg/L who had been on treatment for 3-5 years. One of them underwent a change in their treatment plan from oral alendronate to IV zoledronate when their memory function deteriorated during a follow-up assessment, and a high risk of falls was identified. The oral alendronic acid regimen was unfamiliar to the other two patients, which resulted in poor compliance. However, both patients continued the same treatment plan after implementing appropriate interventions to improve adherence.

We further investigated the reasons for the modification or discontinuation of the initial treatment plan, as illustrated in Figure 4. Treatment was discontinued for five (55.6%) compliant patients who had been taking oral alendronic acid for a minimum of five years when P1NP levels fell below 35 µg/ml (Figure 4D). We also identified a group of four (11.8%) patients who were transitioned from bisphosphonates to teriparatide treatment due to deteriorating BMD as indicated by DXA scan results (Figures 4b-4d). This transition occurred after the completion of an IV zoledronic acid treatment course or four or more years of oral alendronic acid. The P1NP level was less than 35 ug/ml in all of these patients. Additional causes were identified, such as a second fragility hip fracture in a patient who was already receiving bisphosphonate treatment. Finally, we identified three patients who necessitated a transition from oral alendronic acid to subcutaneous denosumab as a result of suboptimal creatinine clearance during subsequent follow-up; two of them had suppressed P1NP levels (Figure 4D), while the other had a high P1NP level of more than 40 µg/ml (Figure 4B). 

## Discussion

In the current study, we investigated the role of pre-operative P1NP measurement in older patients who sustained fragility femoral neck fractures while receiving osteoporosis treatment and explored its association with subsequent bone health management decisions documented after the index admission. Our findings showed that 58.6% of patients with suppressed P1NP levels had a documented change in management, most commonly among those who had been on treatment for over five years. The International Osteoporosis Foundation (IOF) and the European Calcified Tissue Society (ECTS) Working Group advise that a significant decrease in P1NP in the short term supports continuation of treatment [15]. In routine practice, decisions about whether to continue, modify, or discontinue osteoporosis therapy after a fracture are multifactorial; in selected cases, P1NP results may be considered as one component of this assessment alongside DXA and the broader clinical context.

In the present study, we identified 18 patients (31%) who had a P1NP level of 40 µg/L or higher, indicating unsuppressed bone turnover. Several factors may contribute to elevated P1NP levels, including inadequate medication absorption, poor adherence to treatment, or concomitant conditions that impact bone metabolism [16]. Identifying and addressing these factors may help optimise post-fracture bone health planning and support appropriate interventions. However, Gillett et al. indicated that a transient increase in P1NP associated with a decrease in CTX may indicate effective uncoupling and efficacy [6,17]. In our cohort, three (16.7%) of these 18 patients had their treatment plans altered, frequently in the context of concerns such as poor adherence or deteriorating memory function.

Furthermore, our research highlights the possible use of P1NP as a supplementary tool to complement DXA scan findings and other clinical criteria when making therapy choices. For instance, we identified four (11.8%) patients who were transitioned from bisphosphonates to teriparatide treatment due to deteriorating BMD as indicated by DXA scan results, even though their P1NP levels had been suppressed. This underscores the significance of considering a variety of factors when managing patients with fragility fractures and osteoporosis. 

Several limitations should be emphasised. First, this was a retrospective, single-centre, descriptive study and therefore cannot establish causality between P1NP results and clinical decisions. Second, the sample was small and highly selected because P1NP testing was not routine; inclusion depended on whether clinicians requested the test, introducing selection bias and limiting generalisability. Third, there was no comparator group (e.g., similar patients without P1NP testing), and baseline pre-treatment P1NP or consistent DXA data were not available for all patients, restricting interpretation of treatment response. Fourth, management decisions were derived from routine clinical documentation and may be subject to documentation bias; where P1NP was not explicitly referenced, attribution is uncertain. Finally, we did not assess downstream outcomes (e.g., subsequent fractures, BMD change, adverse events, mortality), so the impact of incorporating P1NP into decision-making on longer-term clinical outcomes was not assessed in this study. These limitations require that the findings be interpreted within the context of a retrospective descriptive design, while still providing insight into real-world practice.

Overall, this study describes the use of P1NP testing in a clinically important subgroup, patients who fracture while already receiving osteoporosis therapy, and illustrates how P1NP results may be considered alongside DXA and other clinical factors when informing post-fracture bone health plans. 

## Conclusions

Our findings indicate that the measurement of P1NP may contribute useful context to the formulation of patient-centred decisions regarding the treatment of osteoporosis and fragility fractures and that it was considered alongside DXA findings and the broader clinical context during MDT post-fracture bone health planning. In particular, unsuppressed P1NP was observed in a subset of patients in whom potential contributory factors (e.g., adherence, absorption, comorbid conditions) were considered, while suppressed P1NP commonly coincided with documented decisions to stop or adjust therapy in long-term treated patients (>5 years). However, due to the retrospective design, small and selected cohort, lack of a comparator group, and absence of outcome data, the findings should be interpreted within these methodological constraints; nevertheless, they provide practical, real-world insight into current MDT use of P1NP in this clinically important subgroup. Further evaluation in broader cohorts would help clarify in which patients P1NP testing is most informative and support development of a more standardised approach to its use within post-fracture bone health pathways.
